# Tricolorin A as a Natural Herbicide 

**DOI:** 10.3390/molecules18010778

**Published:** 2013-01-09

**Authors:** Blas Lotina-Hennsen, Beatriz King-Díaz, Rogelio Pereda-Miranda

**Affiliations:** 1 Departamento de Bioquímica, Facultad de Química, Universidad Nacional Autónoma de México, México D.F., C.P. 04510, Mexico; E-Mail: kingbeat@unam.mx; 2 Departamento de Farmacia, Facultad de Química, Universidad Nacional Autónoma de México, México D.F., C.P. 04510, Mexico; E-Mail: pereda@unam.mx

**Keywords:** germination, *Lolium mutliflorum*, *Physalis ixocarpa*, pre-emergence herbicide, post-emergence-herbicide, photosynthesis, seed respiration, *Trifolium alexandrinum*, *Triticum vulgare*

## Abstract

Tricolorin A acts as pre- and post-emergence plant growth inhibitor. In pre-emergence it displays broad-spectrum weed control, inhibiting germination of both monocotyledonous (*Lolium mutliflorum* and *Triticum vulgare*) and dicotyledonous (*Physalis ixocarpa and Trifolium alexandrinum*) seeds, being the dicotyledonous seeds the most inhibited. Tricolorin A also inhibited seedling growth, and seed respiration, and since the concentrations required for inhibiting both germination and respiration were similar, we suggest that respiration is one of its targets. Tricolorin A at 60 µM acts as a post- emergence plant growth inhibitor by reducing dry plant biomass by 62%, 37%, 33%, and 22% for *L. multiflorum*, *T. alexandrinum*, *T. vulgare*, and *P. ixocarpa*, respectively, 18 days after its application. In order to determine the potency of tricolorin A as a plant growth inhibitor, paraquat was used as control; the results indicate that tricolorin A acts as a non-selective post-emergence plant growth inhibitor similar to paraquat, since both reduced the biomass production in *P. ixocarpa* and *T. alexandrinum.* Therefore, we suggest that *tricolorin A* will be a good biodegradable herbicide for weeds.

## 1. Introduction

The widespread use of synthetic herbicides to manage weeds has resulted in herbicide-resistant weeds and in addition public concerns over the impact that synthetic herbicides may have on human health and in the environment are increasing [1,2,3]. These concerns are shifting attention to alternative weed control technologies based on natural products [1,3]. Many natural products inhibit plant growth, and some of these are allelochemicals. The size of this category of natural products depends upon the definition of allelopathy [4]. Allelopathy is a controversial word that has existed within the scientific community for the last four decades, however it has still not been acknowledged by many scientists. According to the definition given by the International Allelopathy Society (IAS), allelopathy ‘studies any process involving secondary metabolites produced by plants, algae, bacteria and fungi that influence the growth and development of agricultural and biological systems [5]. However, allelopathy appears to be an important component of plant interference capability in a variety of natural ecosystems [6].

**Figure 1:**
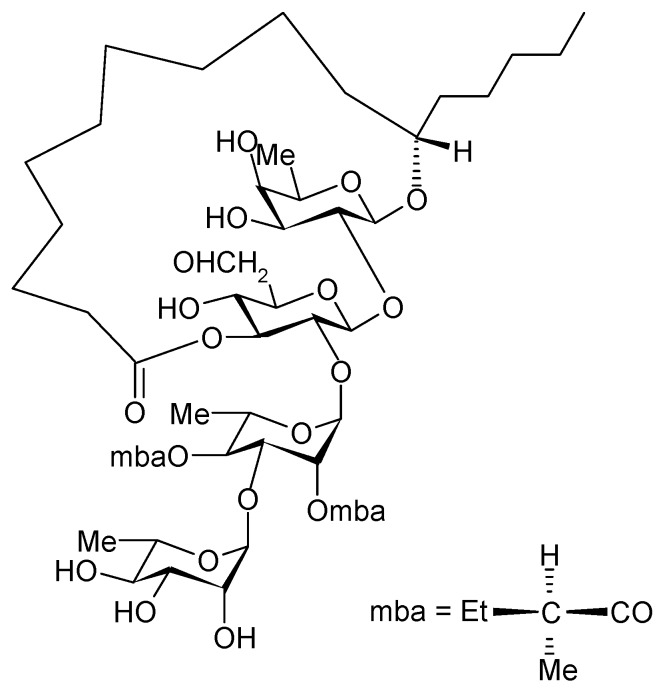
Struture of Tricolorin A.

The allelopathic nature of plants of the genus *Ipomoea* (Convolvulaceae) shows suppressive effects on the growth of weeds of sweet potatoes, *Ipomoea batatas* (L.) Lam. [7,8]. The joint cultivation of some semi-domesticated legumes, together with some *Ipomoea* species, is a common agricultural practice in Mexico to minimize the growth of an associated weed [9]*.* Farmers in the state of Morelos (México) use *Ipomoea tricolor*, which are toxic to weeds, as a cover crop during the fallow period in sugar cane fields. A study thatcombined Petri dish assays with greenhouse experiments suggested that the suppressive activity of *I. tricolor* may involve both leaching of allelochemicals from the living plants by rain and release of allelochemicals from decaying plant matter [10]. Pereda Miranda *et al.* [11] isolated tricolorin A from *Ipomoea tricolor*, and elucidated its structure as (11*S*)-hydroxy-hexadecanoic acid 11*-O-α-*L*-*rhamnopyranosyl-(1→3)-*0*-α-L-[2-*0*-(2*S*-methylbutyryl)-4-*0*-(2*S*-methylbutyryl)] rhamnopyranosyl-(1→2)-*O*-β-D-glucopyranosyl-(1→2)-β-D-fucopyranoside-(1,3"-lactone) (Figure 1). 

The effect of tricolorin A on seed germination of *E. crus-galli* and *A. leucocarpus* was investigated, finding that it inhibited both with IC_50_ values of 36.1 µM and 149.4 µM, respectively, and that it also inhibited root elongation in both species with IC_50_ values of 12.3 µM and 36.2 µM, respectively [11]. The concentration threshold required for most of the natural products that inhibit plant growth tested in similar experimental designs is often in the range of 100–1000 µg/mL [12], therefore, tricolorin A has shown a stronger inhibitory effect on various weeds and it had higher potency than other compounds.

Continuing with the characterization of tricolorin A as an inhibitor of the light reaction of photosynthesis, we found that at low concentrations it acts as a potent uncoupler, with an UC_50_ (concentration required for 50% of enhancement of basal electron flow) value of 0.33 µM in spinach chloroplasts, but at higher concentration it behaved as a Hill reaction inhibitor with an IC_50_ value of 5 µM. Our chlorophyll *a* fluorescence analysis results showed that tricolorin A induced accumulation of Q_A_^−^ species and strongly decreased the electron transport capacity, indicating that the target of this molecule was located at the Q_B_ level [12]. When the macrocyclic lactone of tricolorin A was broken by saponification, the resulting glycosidic acid form lost its activity, thus its intact macrocyclic structure is important for its bio-activity [11]. The present work represents a contribution to the research on the genus *Ipomoea* (Convolvulaceae) that could support the development of “green herbicides”. We investigated some of the characteristics of tricolorin A as a herbicide for weeds. For this propose both monocotyledonous and dicotyledonous plants were used in assays to determine the potential selectivity of this natural product. We found that tricolorin A acts as pre- and post-emergent herbicide. 

## 2. Results and Discussion

### 2.1. Pre-Emergence Bio-Assay Effect on Seed Germination and Seedling Growth

**Figure 2:**
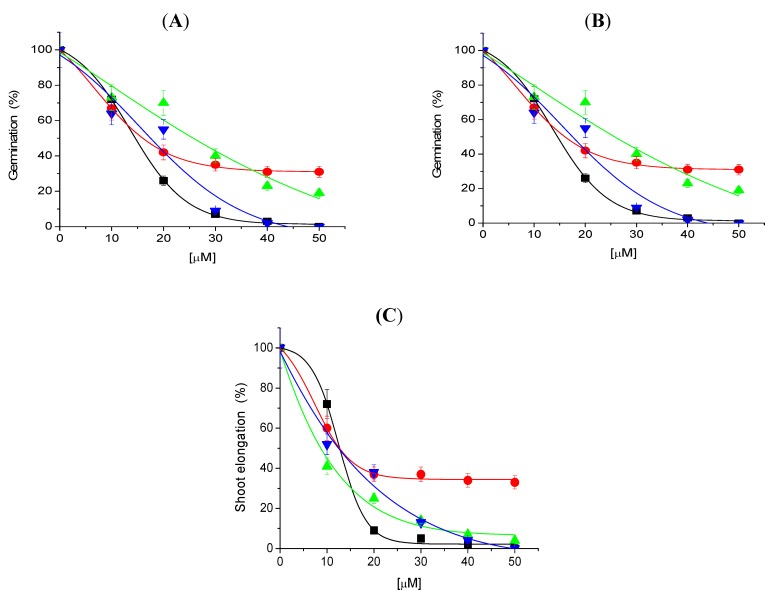
Effect of tricolorin A on germination (**panel A**), root (**panel B**) and shoot (**panel C**) elongation for all seedlings tested. The following color are used for *P. ixocarpa* (black symbols), *T. alexandrinum* (red symbols), *T. vulgare* (green symbols) and *L. mutliflorum* (blue symbols). All the parameters are presented as percentage. Non treated seeds were used as control and was taken as 100% of germination or root and shoot elongation. Data are average of three replicates. The bars represent the maximum standard deviations (See Experimental).

Tricolorin A inhibited seed germination of *Physalis ixocarpa*, *Trifolium alexandrinum*, *Lolium mutliflorum* and *Triticum vulgare*, in a dose-dependent pattern (Figure 2).

**Figure 3:**
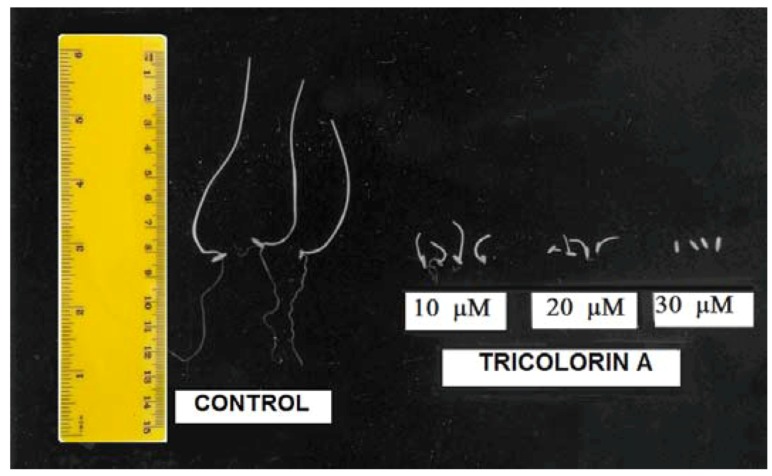
Effect of tricolorin A on root and shoot length of *L. multiflorum* seedlings.

*P. ixocarpa* and *L. multiflorum* (Figure 2A–C and Figure 3) were the most sensitive species since 100% inhibition was observed with 50 µM of tricolorin A. Root and shoot elongation were inhibited on all tested plants by tricolorin A in a concentration dependent manner (Figure 2). Figure 3 illustrates the inhibition of root and shoots length on *L. multiflorum* seedlings produced by 30 µM of tricolorin A. 

IC_50_ values for seed germination of *P. ixocarpa*, *T. alexandrinum*, *L. mutliflorum* and *T. vulgare* in the presence of tricolorin A calculated from Figure 2 were 13.8, 16.0, 16.1 and 25.7 µM, respectively. Germination of *T. vulgare* was the least affected (Figure 2B). 

The inhibitory effects of tricolorin A on the elongation of roots and shoots of monocotyledonous and dicotyledonous are shown in Table 1 as IC_50_ values. Roots of *T. vulgare* were the least affected by triclorin A, and in all seedlings and shoots were more affected by tricolorin A than roots. 

Researchers on allelopathy studies of germination and seedling growth inhibition with natural products, *i.e.*, Anaya *et al.*, studied the root development of *Amaranthus leucocarpus* and *Echinochloa crusgalli* inhibition with organic extract of *I. tricolor* and found glicosidic resin was the most active root development inhibitor [7].

**Table 1:** Effect of tricolorin A on seedling growth.

	IC_50_ (µM)	
	Root	Shoot
Dicotyledonous plants		
*P. ixocarpa*	13.8	12.7
	*p* = 8.6 × 10^−4^	*p* = 2.55 × 10^−6^
*T. alexandrinum*	18.0	12.7
	*p* = 1.6 × 10^−2^	*p* = 5.7 × 10^−2^
Monocot plants		
*T. vulgare*	24.1	8.5
	*p* = 1.37 × 10^−6^	*p* = 7.2 × 10^−7^
*L. multiflorum*	15.3	12.3
	*p* = 8.02 × 10^−7^	*p* = 6.5 × 10^−5^

The IC_50_ values (were obtained from the Figure 2) for each one is the concentration that produces 50% inhibition and was obtained for all the measurements of roots and shoots elongation. The T student test for two populations was used as statistical analysis to determine if the difference between control values and assays values were significant, the *p* values were < at 0.05, its means the differences were significant.

Sorgoleone, a p-benzoquinone isolated from *Sorghum bicolor* root exudate inhibits the germination and growth of susceptible weeds at concentrations as low as 10 µM [13,14]. Indeed, sorgoleone concentrations in soil under sorghum crops can easily reach 10–100 µM [15]. This compound has a variety of effects on plant metabolism, including inhibition of photosynthesis and respiration [16]. Almost all other natural product concentration threshold required to inhibit plant growth tested in similar experimental designs is often in the range of 100–1,000 µg/mL [12]. 

### 2.2. Effect of Tricolorin A on Seed Respiration

**Figure 4:**
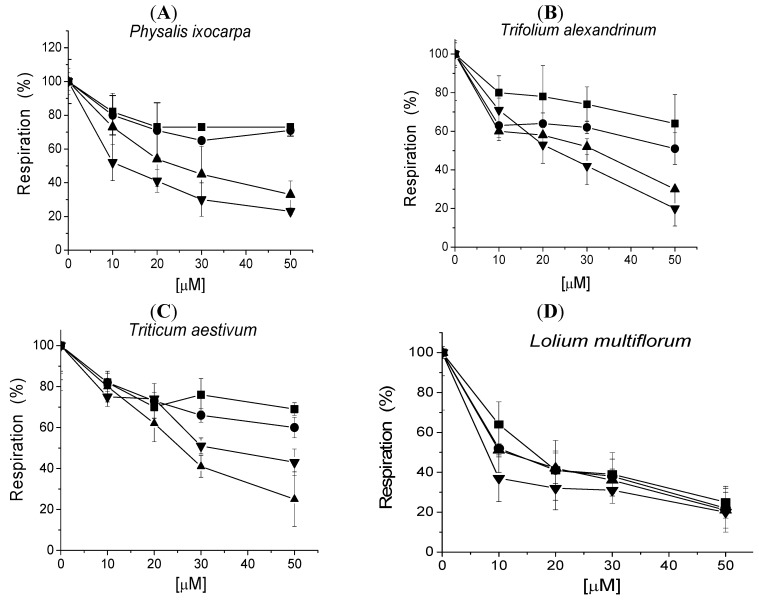
Effect of increasing concentrations of tricolorin A on seed respiration. The seeds were previously imbibed for 5 (■), 24 (●), 48 (▲) and 72 (▼) h and then seed respiration was recorded. Experiments were conducted in replicates and data are expressed as the means ± S.E. (standard errors), obtained with the software Origin 6.0.

In order to know which primary metabolism is affected by tricolorin A, seed respiration was measured during germination after 5, 24, 48 and 72 h of imbibitions with water and the O_2_ uptake was used to measure respiration. Figure 4 show that the respiration of the four seeds tested was inhibited. The seed respiration inhibition increases as time of imbibitions increases and also seed respiration inhibition increases as concentration from 10, 20, 30, and 50 µM of tricolorin A increases (Figure 4).

In most cases, respiration was inhibited with tricolorin A in a concentration (from 10, 20, 30 and 50 µM) and time dependent manner. The IC_50_ values calculated for each species at 72 h of imbibitions, with only 50 µM tricolorin A were 8.0, 11.7, 22.6 and 25.7 µM for *L. mutliflorum*, *P. ixocarpa*, *T. alexandrinum* and *T. vulgare*, respectively. Since tricolorin A affects seed germination, root ant shoot development and also respiration during seedling, this suggests that it has more than one target. Another natural product that affects respiration and photosynthesis like tricolorin A is sorgoelone. Both tricolorin and sorgoleone inhibit the light reactions of photosynthesis both compounds interact and inhibit at Q_B_ level [17].

When tricolorin A affects respiration in mitochondria it acts as an uncoupler and its behavior is similar to that of other natural products, like juglone, a phenolic compound [18] or the anacardic acids, other natural products found in cashew nut shell oil, the nut and fruit juice exhibited uncoupling effects [19]. Thyrsiferol selectively suppressed mitochondrial respiration at complex I (IC_50_ 3 μM) but has a rotenone-like activity that may contribute to the observed cytotoxicity and play an important role in *Laurencia* chemical defense [20]. 

### 2.3. Effect of Post-Emergence Bio-Assay on Dry Biomass

To know if tricolorin A acts as a post-emergent herbicide, plants were grown for 18 days in the greenhouse, after this time they were sprayed with increasing concentrations of tricolorin A and paraquat. Fourteen days after the application, their effects were evaluated by measuring the dry biomass of all leaves of plants tested. *Physalis ixocarpa* plants were less affected by both compounds (22% and 16% of inhibition with *tricolorin A* and paraquat, respectively) compared to *Trifolium alexandrinum* (37% and 43%). Furthermore, *T. alexandrinum* was more affected with paraquat (Table 2) than with tricolorin A. Visual observations showed that tricolorin A caused necrosis on the leaves of *P. ixocarpa* and *T*. *alexandrinum* plants. Table 2 shows that the two studied monocotyledonous plants were affected as concentrations of both tricolorin A and paraquat. For example, *T. vulgare* was the less affected of the two, the inhibition with tricolorin A 60 µM was 62% (Table 2), while the dry biomass of the aerial parts of the grass *L. multiflorum* decreased at increasing concentrations of tricolorin A, *i.e.*, at 60 µM by 68% (Table 2). Therefore, both tricolorin A and paraquat acts as a non-selective post-emergence plant growth inhibitor since both compounds reduced the biomass production in all plants tested (68%) (Table 2).

**Table 2:** Effect of tricolorin A on total dry biomass of plants of *P. ixocarpa*, *T. vulgare*, *L. mutliflorum* and *T. alexandrinum*. Plants were grown for 18 days before tricolorin was sprayed on leaves.

	Tricolorin A		Paraquat	
**Conc.µM**	Dry biomass (g)	%	Dry biomass (g)	%
*Physalis ixocarpa*				
0	0.446 ± 0.025	100	0.446 ± 0.025	100
20	0.428 ± 0.027	96	0.464 ± 0.029	104
40	0.375 ± 0.044	84	0.446 ± 0.045	100
60	0.348 ± 0.035	78	0.375 ± 0.065	84
*Trifolium alexandrinum*				
0	0.486 ± 0.036	100	0.486 ± 0.036	100
20	0.423 ± 0.042	87	0.325 ± 0.032	67
40	0.403 ± 0.093	83	0.287 ± 0.048	59
60	0.306 ± 0.088	63	0.277 ± 0.052	57
*Triticum vulgare*				
0	1.875 ± 0.084	100	1.875 ± 0.084	100
20	1.562 ± 0.285	82	1.562 ± 0.269	83
40	1.47 ± 0.3174	78	1.250 ± 0.176	67
60	1.250 ± 0.156	67	1.250 ± 0.219	67
*Lolium multiflorum*				
0	1.783 ± 0.266	100	1.783 ± 0.266	100
20	1.622 ± 0.149	91	1.729 ± 0.195	97
40	1.123 ± 0.096	53	0.963 ± 0.149	54
60	1.034 ± 0.137	38	0.571 ± 0.058	32

Experiments were conducted with 3 replicates and data were expressed as means ± S.DE (standard errors), obtained with the program Origin 6.0.

## 3. Experimental

### 3.1. Seed Germination Bioassays

*Lolium multiflorum*, *Triticum. vulgare* var. Salamanca, *Trifolium alexandrinum* and *Physalis ixocarpa* seeds were purchased from Semillas Berentsen S. A. de C. V. (Celaya, Guanajuato, Mexico). For these experiments the seeds were selected for uniformity, the damaged ones were discarded, and 50 seeds of *L. multiflorum*, *T. alexandrinum*, and *P. ixocarpa* and 20 seeds of *T. vulgare* were placed on filter paper (Whatman No. 1) in Petri dishes (85 mm diameter). The number of seeds was selected in accord to their size. In three replicate experiments, the paper was wetted with 10 mL of de-ionized water or test solution (the compound tricolorin A was dissolved in MeOH, the concentration of MeOH was less than 1% and paraquat was dissolved in deionized water). The dishes were wrapped with Parafilm and incubated at 28 °C in the dark and percentages of germination and root lengths were obtained after five days for *L. multiflorum*, *T. alexandrinum*, and *P. ixocarpa* and four days for *T. vulgare* because this seed required less germination time than the other seeds*.* The number of germinated seeds was determined according to the criteria of 1 mm extrusion of the root. Root and shoot length were measured for all seeds germinated. Statistical differences between the treatments were evaluated by the Student’s t-test for assessing the statistical significance of the difference between two sample means. Control seed dishes contained the same amount of seeds, volume of water, and methanol (less than 1%) as the test solutions.

### 3.2. Seed Respiration

To measure the effect of tricolorin A on seeds respiration, the same procedure as that for germination bioassay, however, 300 seeds of *L. multiform* and *P. ixocarpa*, 50 seeds of *T. alexandrinum*, and 20 seeds of *T. vulgare* were placed in 10 cm Petri dishes. The number of seeds used for each experiment was the one that produce a measurable level of O_2_ uptake. The seed respiration was measured as O_2_ uptake using a Clark type electrode attached to a Yellow Spring Instrument (YSI) model 5300A oxymeter. The current generated during O_2_ reduction to water was converted to voltage, and the signal recorded on a Kipp and Zonen chart recorder. The current was stoichiometrically related to the oxygen consumed at the cathode. The seeds were imbibed in 10 mL of water on Petri dishes, different concentrations of tricolorin A were added ranging from10 to 50 µM, after 5, 24, 48 and 72 h the O_2_ uptake was measured, and the consumption rate was calculated with the follow equation:

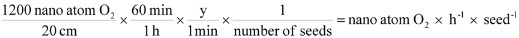

and reported in nano atom O_2_ × h^−1^ × seed [21], where 1,200 nano atom O_2_ corresponded to 20 cm in the recorder paper and “*y*” are the recorded distances in cm when the O_2 _uptake of seeds in aqueous medium occurred. 

### 3.3. Post-Emergence Bioassays

The seeds of two monocotyledonous species (*Lolium multiflorum* and *Triticum. vulgare*) and two dicotyledonous species (*Physalis ixocarpa* and *Trifolium alexandrinum*) were germinated as in the seed germination assays. After four days the seedlings were transplanted into 12-cm diameter pots containing a 2:1 mixture of soil-sand. All pots were irrigated daily and maintained near to field capacity. In the greenhouse, T = 25–30 °C with natural day/night illumination. After 18 days of emergence plants of similar size of dicotyledonous and monocotyledonous species were divided into two groups: the control and the experimental. The experimental plants were sprayed manually with 20, 40 and 60 µM tricolorin A prepared as an aqueous suspension containing maize oil (0.1% w/v, as adjuvant) [22] and 0.25% v/v Tween 20 (added to reduce the surface tension of the suspension). The control group was sprayed with distilled water containing the same amount of MeOH used to dissolve each aliquot of tricolorin A to prepare each of the concentrations, Tween 20, and maize oil [23] and fourteen days after compound was sprayed the dry biomass was weighed. It is well known that paraquat (*N*,*N*′-dimethyl-4,4′-bipyridinium dichloride or methylviologen) is a contact herbicide, with a molecular target at the photosystem I in the chloroplast, diverting electrons from the iron–sulfur centers [24]. To know the potency of tricolorin A and its selectivity we used paraquat as positive control in the same concentration as tricolorin A. The IC_50_ (or CU_50_) for all activities tested was calculated by linear regression analysis indicated a concentration value required to cause 50% inhibition, and were obtained by probit analysis [25].

## 4. Conclusions

Tricolorin A acted as pre- and post-emergence herbicide. As a pre-emergent herbicide it showed broad-spectrum weed control, since inhibited both monocotyledonous (*Lolium mutliflorum* and *Triticum vulgare*) and dicotyledonous (*Physalis ixocarpa* and *Trifolium. alexandrinum*) seed germination. *P. ixocarpa* and *L. multiflorum* were the most inhibited and *T. alexandrinum* was the less affected. Tricolorin A inhibited seedling growth and seed respiration in the same concentration dependent manner. Tricolorin A at 60 µM acted also as a non-selective post-emergent weed growth inhibitor since 18 days after its application the dry biomass was reduced for all plants studied; this represents the first report of tricolorin acting as a post-emergent herbicide. We have previously demonstrated that *tricolorin A* inhibits photosynthesis *in vitro* [12] at the same concentration that decreased biomass production; therefore, we suggest that inhibition of photosynthesis was one of the targets of tricolorin A when it acted as a post-emergent herbicide and this may be the explanation for the growth reduction caused by tricolorin A. Together, the results of the present work pointed to the fact that tricolorin A is a good candidate for the development of new, biodegradable, and environmentally safe herbicides. The IC_50_ values that had been previously reported for tricolorin A on germination were 36.1 µM to 149.4 µM [11], while published IC_50_ values of most of the natural products proposed as herbicides vary in the range of 100-1000 µg/mL [13], thus making tricolorin A one of the most active natural products in this area.
